# Basic personal values in the midst of the COVID-19 pandemic in Italy: A two-wave longitudinal study

**DOI:** 10.1371/journal.pone.0274111

**Published:** 2022-09-09

**Authors:** Michele Vecchione

**Affiliations:** Department of Social and Developmental Psychology, Sapienza University of Rome, Rome, Italy; University of Rome, ITALY

## Abstract

This study investigated value change during two phases of the COVID-19 pandemic in Italy, one of the most affected countries in the world. The first wave of data was collected in summer 2020, when the virus was on the retreat. The second wave was collected in autumn, at the peak of the second pandemic wave (November 2020). We investigated how Schwartz’s higher-order values changed over the two waves of the study, using economic condition as a predictor of change. We also examined whether value change predicted subsequent value-expressive behavior. Results showed no mean-level change for self-enhancement, self-transcendence, conservation, and openness to change values, but significant interindividual differences in the amount of change for each of the four values. Economic condition emerged as a significant predictor of change in conservation values: Individuals with a decreasing income since the beginning of the pandemic were more likely to increase the importance assigned to these values with respect to individuals whose economic well-being has remained unchanged. Moreover, an increase in conservation and openness to change values predicted behaviors that are mostly expressive of these values, above and beyond value importance at Time 1. Results and their implications for the study of values are discussed.

## Introduction

Basic personal values are enduring beliefs concerning desirable goals that operate as guiding principles in people’s lives [1–3]. Although values are generally consistent across time, they may encounter significant modifications over an individual’s life course [4, 5]. Bardi and Goodwin [6] argued that the adaptation to changing life conditions is one of the most prominent facilitator of value change [7]. Several studies have found support for this notion, showing that personal values can be shaped by a number of critical life events, such as the diagnosis of serious health adversities [8], the 2006 Israeli Lebanese war [9], the migration toward other countries [10, 11], the September 11 terrorist attacks [12], and a military mission in Afghanistan [13].

The present study fits into this line of inquiries by investigating value change during the COVID-19 outbreak, an unprecedented event in recent history that has profoundly changed the daily lives of millions of people around the world. Besides the immediate effects on health, the pandemic has affected people’s habits, their material and psychological conditions, the way they relate to others [14, 15]. This might prompt people to reconsider what is important in life.

**Fig 1:**
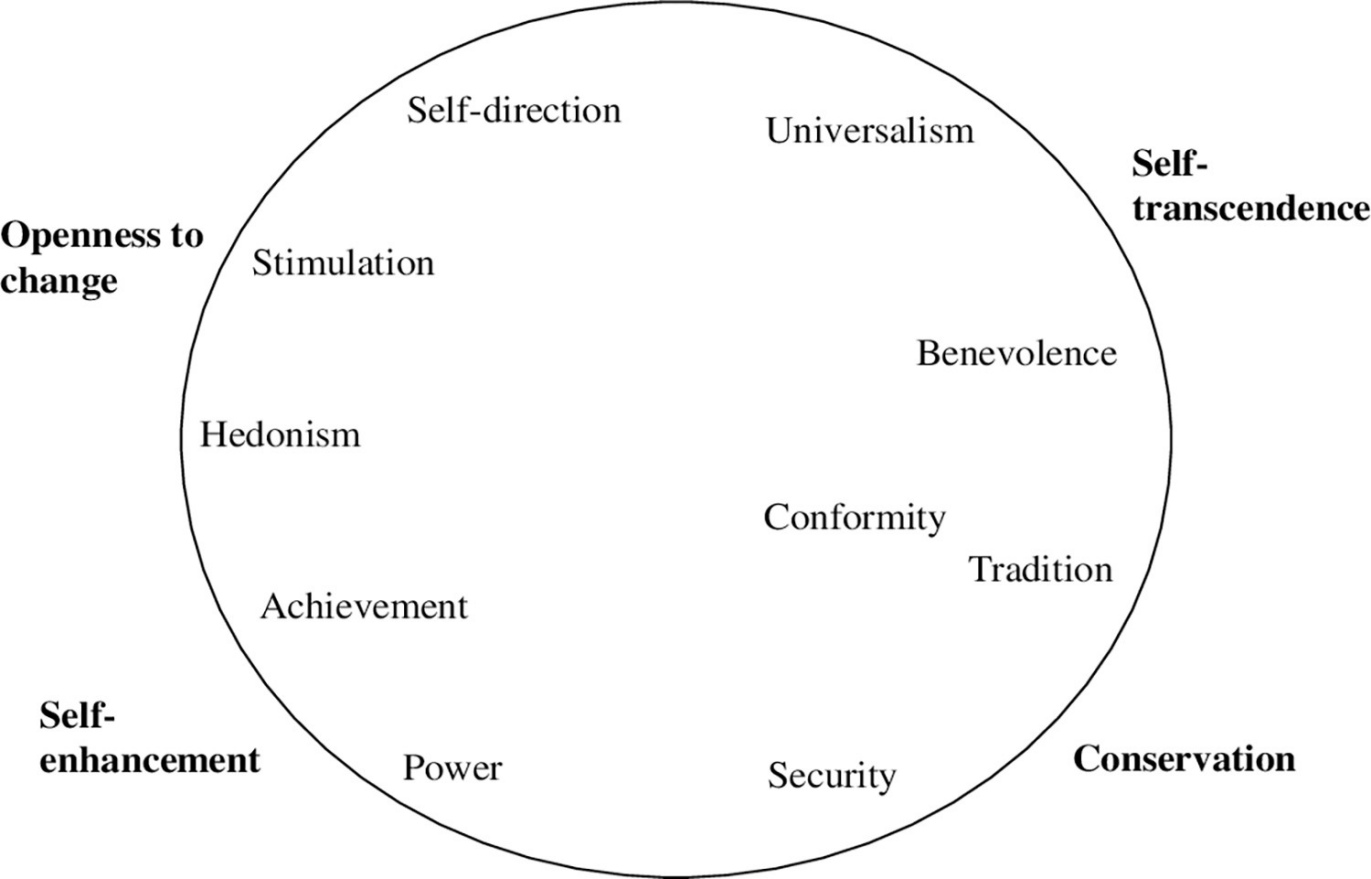
Theoretical model of relations among ten basic values (Schwartz, 1992).

In our investigation, we adopted the Schwartz theory of basic human values. In its classic form [3], the theory identifies 10 motivationally distinct values: security, conformity, tradition, benevolence, universalism, self-direction, stimulation, hedonism, achievement, and power (see Fig 1). A refinement of the theory [16] has recently suggested an alternative partitioning of the motivational continuum into 19 more narrowly defined value constructs. The 10 original values, as well as the 19 values of the refined theory, can be grouped into four higher order dimensions. Self-enhancement values (achievement, power) promote success and dominance over others. These values conflict with Self-transcendence values (universalism, benevolence), which nurture promoting the welfare of others. Conservation values (conformity, tradition, security) emphasize self-restriction, preservation of the past, stability, and predictability. They conflict with Openness to change values (self-direction, stimulation, hedonism), which encourages autonomy and receptiveness to change [3, 17].

Researchers have started to address the question of whether and how the COVID-19 pandemic can affect the individual’s value priorities. A first longitudinal study by Daniel et al. [18] examined value change before and during the pandemic (from 2017 to December 2020) using data available from the Values Project, a large online survey conducted in Australia. Values were measured using the Schwartz values best–worst survey (SVBWS) [19]. Findings have shown that conservation values increased in importance after the onset of the global COVID-19 crisis (April 2020) and slightly decreased by the end of 2020. Openness to change values decreased in importance but returned to the initial level in late 2020. Self-transcendence values decreased progressively after the pandemic. Of interest, the observed changes were more pronounced among individuals with high levels of worry about SARS-CoV-2 infection.

A second longitudinal study was conducted in Poland by Bojanowska, Kaczmarek, Koscielniak and Urbańska [20]. The study used the PVQ-57 [16] for assessing the 19 values of the refined theory at three measurement occasions: nine months before the first lockdown in Poland, in June 2019 (T1), two weeks into the first lockdown (T2) and two weeks afterwards (T3). The results have shown that conformity–rules, humility, universalism–nature, universalism–tolerance, and self-direction–though increased from T1 to T3, while hedonism decreased. Other values (i.e., personal and societal security, conformity–interpersonal, benevolence–caring, and universalism–concern) increased from T1 to T2, but the increase tended to disappear at T3.

A cross-sectional study by Bonetto et al. [21] compared participants’ values in usual life with their values during the first French lockdown period (i.e., between April and May 2020). The same group of respondents completed two versions of the short form of the Portrait Values Questionnaire (PVQ-21) [22], which varied according to the response context (i.e., values usually in life vs. during the Covid-19 outbreak). The data revealed that conservation values were higher than usual when referred to the outbreak. The opposite result was observed for self-enhancement and openness to change values.

The above results provide important insights on the effect that the pandemic had on people’s value orientations. However, a number of important differences, such as the timing of the data collection and the cross-country variability in the severity of the pandemic, make the results from different studies not directly comparable. More data are needed before firm conclusions can be drawn. The current study intends to contribute to this body of research by examining value change during two different phases of the COVID-19 pandemic in Italy, one of the most affected countries in the world [23]. We first provide a brief description of the Italian context in which the study was occurred. We then presented the aim of study.

### The Italian case

The first infection by SARS-CoV-2 in Italy was registered on February 20, 2020. The number of infections increased dramatically in the subsequent few weeks, and Italy rapidly became the epicentre of the pandemic in the world. The peak of the ascending curve was reached in March, when the country entered lockdown. The epidemiological situation started to improve in May 2020, with a progressive drop in infections, hospitalizations, and deaths. A further improvement was observed throughout the summer [24, 25], when the pandemic appeared to have been brought under control and the restrictions that were in place over the spring were gradually eased. During the second half of August, however, a new increase in the number of cases was observed. This increase was the prelude of a second, even more severe pandemic wave, which started in the fall. This wave reached its peak at the end of October 2020 [26], when the overwhelm of the health systems brought the Italian government to introduce a number of unprecedented restrictive measures in the attempt to contain the spread of the infection [27].

### The current study

Using a two-wave longitudinal design, the current study examined value change over different phases of the COVID-19 outbreak in Italy. The first wave of data was collected in the midst of the summer 2020 (end of July), while the second took place about 100 days after, in November 2020. At first, we examined mean-level change in the four higher order values over the three months of the study. Personal values are generally quite consistent over such relatively short periods of time [28, 29]. However, the two measurement occasions depict distinct phases of the pandemic scenario in Italy. At time 1, the virus was clearly on the retreat. The number of daily new cases had dropped to the lowest levels since the beginning of the pandemic and the containment measures were almost totally released. This has led some authorities of the Italian healthcare system to argue that the virus “clinically no longer exists” [30]. Time 2, by contrast, correspond to the peak of the second and worst wave of the pandemic. On the 4 November 2020, the Italian prime minister Giuseppe Conte described the situation as particularly critical and announced a new lockdown [31]. It is likely that, under such aversive conditions, individuals may adjust their values in order to fit the changing environment. Conservation values express self-protective, anxiety-control orientations [5, 32]. These values gain importance and are more likely to be activated under threatening circumstances [33]. We may therefore expect an average increase in conservation values from the first to the second wave of data.

In accordance with the pattern of conflicts and compatibilities predicted by Schwartz’s model [3], we also expected a concomitant decrease in openness to change values. The introduction of stringent containment measures in response to the increased severity of the pandemic, along with the heightened social pressure to behave in expected ways, was likely to restrict opportunities for the expression of values that emphasize personal autonomy, self-gratification, excitement, and novelty.

The mean level, however, provides only limited information about the process of change. We therefore extended the analysis of value change at the intraindividual (i.e., the within-person) level. First, we examined the amount of interindividual differences in how values vary within the person. We expected a significant variability around the average change. Next, we considered potential predictors and outcomes of this variability.

Italy is one of the countries that have experienced the most dramatic economic consequences from the outbreak [34]. The trade-off between public health and economy has been a recurrent theme in the public discourse [35]. We therefore have investigated the role of economic condition as predictor of value change. We expected that individuals who have reported a change in their economic condition during the pandemic were more likely to adjust their value orientations during the two waves of the study. Specifically, conservation values were expected to increase more among individuals with decreasing income with respect to those whose economic well-being improved or remained unchanged. This would accord with earlier research findings showing that the importance of security, tradition and, to a lesser extent, conformity values increased in young people from 16 European countries during the global financial crisis [36].

Moreover, we investigated whether both values and value change predict the individual’s behavior during the COVID-19 outbreak. In accordance with previous research findings [37, 38], we expect that the importance attributed to the four higher order values at T1 would predict behaviors that are mostly expressive of these values. But what about the role of value change? The literature suggests that values that gained importance as a result of changing environmental conditions are likely to become more accessible and thus to increase their predictivity [39, 40]. As Schwartz [41] wrote: “In order for a value to exert influence on an attitude or behavior, it must first be activated. Accessibility increases the probability that a value will be activated, and more important values are more accessible [42]. So important values are activated more often and exert influence” (p. 64). We therefore expected that an increase in the importance assigned to the four higher order values from T1 to T2 would positively predict value-expressive behaviors, above and beyond value importance at T1.

## Materials and methods

### Participants and procedures

A total of 187 individuals agreed to participate in the study. They were briefed on the general aims of the research and provided written informed consent. The study was approved by the ethical committee of the Department of Social and Developmental Psychology (Sapienza University of Rome). Mean age of the sample was 32.95 years (*SD* = 14.26), with 71.0% females. Annual net household income was measured using ten range categories, each corresponding to the deciles of the distribution of Italian household income in 2016. The item was taken from the last wave of the European Social Survey (ESS, round 9, 2018/2019; details on the ESS income measure can be retrieved at: https://www.europeansocialsurvey.org/docs/round9/survey/ESS9_appendix_a2_e03_0.pdf). Participants were asked to select the category that better describes their household’s total income, after tax and compulsory deduction. Responses were distributed as follows: 11.2% “less than 9.000 Euro”, 12.3% “from 9.000 to 14.000”, 11.8% “from 14.001 to 17.500”, 11.8% “from 17.501 to 21.000”, 12.8% “from 21.001 to 25.000”, 8.6% “from 25.001 to 29.500”, 10.7% “from 29.501 to 36.000”, 10.2% “from 36.000 to 43.500”, 5.3% “from 43.500 to 56.000”, 5.3% “more than 56.000 Euro”. A chi-square goodness of fit test showed non-significant differences between the observed distribution and the distribution obtained from the Italian sample of the ESS (round 9): χ^2^(9) = 15.492, p = .078 (analysis were performed by using the anweight–analysis weight–variable as a weight in the ESS data. This corrects for differential selection probabilities: https://www.europeansocialsurvey.org/docs/methodology/ESS_weighting_data_1_1.pdf).

Data were collected via the Qualtrics online platform. At time 1 (July 2020), participants completed a shortened version of the Portrait Values Questionnaire that includes 21 of the original 40 items (PVQ-21) [22]. The same instrument was completed again at time 2 (November 2020). In this wave of the study participants were also asked to provide information on their own feelings about economic condition and to report on their own behaviors during the previous three months. Multivariate analysis of variance showed that the mean scores on the four higher order values at T1 were not significantly different between participants who provided complete data (83% of the sample) and those who did not: F(4,164) = 0.62, p = .65.

### Measures

#### Basic values

We measured values with the PVQ-21 [22]. Each item comprises a short description of a person’s goals, wishes or aspirations that implicitly suggest the importance of a value. For example, the item”*It is important to her that the government ensures her safety against all threats*. *She wants the state to be strong so it can defend its citizens*” describes a person for whom security values are important. For each portrait, respondents indicate how similar the person is to themselves on a 6-point scale ranging from “not like me at all” to “very much like me”. Respondents’ values are inferred from the values of the people they consider similar to themselves. We focused on the four broader dimensions (self-enhancement, self-transcendence, conservation, openness to change) because earlier studies conducted with the PVQ-21 on samples from various countries have found a lack of discriminant validity for some of the ten values [43, 44]. In the current sample, Cronbach’s alpha at T1 and T2 were respectively .77 and .72 for self-enhancement, .68 and .66 for self-transcendence, .52 and .53 for conservation, .74 and .72 for openness to change.

#### Behaviors

At time 2 participants completed a brief, 24-item version of the Everyday Behavior Questionnaire (EBQ) [37, 45]. The full-length version comprises 85 items, each describing a specific behavior that mostly express one of 19 value types in Schwartz’s refined theory [16]. The short version used in the present study was developed by selecting 24 items from the original instrument. Respondents were asked to report how often they had engaged in each behavior during the past three months relative to the number of times they had an opportunity to do so, using a 5-point response scale ranging from 1 (never) to 5 (always). Following Schwartz and Butenko [37], we also included the alternative “never had even one opportunity to do something like this”. Responses in this category were treated as missing data, as suggested by the authors [37].

The items of the short form were selected to ensure an appropriate range of content, by covering all the ten basic values of the original theory. Moreover, given the relatively short time of reference for the behaviors (3 months), and considering the restrictions that occurred during the pandemic, we excluded items that could presumably generate a high rate of “never had an opportunity” responses, such as “Participate in an activity aimed at preserving the environment”, and “Go to the movies, theatre, or restaurant just for pleasure”.

Examples of items included are: “Pressure others to go along with my preferences and opinions” and “Put a lot of energy into study, work, sports, music, etc. in order to be successful” for self-enhancement values, “Take care of a friend or family member who was sick”, and “Discuss suffering and poverty in the world with another person” for self-transcendence values, “Take special steps so my family and I avoid getting sick (vitamin or other supplements, wear masks, etc.)”, and “Obey traffic rules even when breaking them would cause no danger” for conservation values, “Pay no attention to outside pressures when making a decision”, and “Look for exciting activities to break up your routine” for openness to change. The alternative “never had an opportunity” was used from 0 to 11% of the time across the 24 items, with a mean percentage of 2.2% (SD = 2.4). The complete list of items with percentages of “never had an opportunity” is available in the S1 Table.

#### Feelings about economic condition

At time 2 participants were also asked to report on their own economic situation, both before and during the pandemic. A first item, taken from the European Social Survey (ESS), was used to enquire into *current* household income. Participants were asked to select, among four alternatives, the one that best described their present condition (percentages of responses are in parentheses): (1) Living comfortably on present income (32.6%), (2) Coping on present income (44.9%), (3) Finding it difficult on present income (18.7%), and (4) Finding it very difficult on present income (3.7%). A second item asked respondents to describe their typical household’s income, as it was *before the pandemic*: (1) Living comfortably (39.0%), (2) Coping (47.6%), (3) Finding it difficult (12.8%), and (4) Finding it very difficult (0.5%). Descriptively, the data revealed an overall worsening of the economic condition from before the pandemic to November 2020 (T2). When looking at the joint frequency distribution, we found that 24% of the sample (*n* = 44) scored lower on the second item (income before the pandemic), thus reporting increasing economic difficulties. The remaining 76% (*n* = 143) did not experience a change. A two-way table reporting the complete pattern of joint frequencies is reported in the S2 Table.

## Statistical analysis

### Measurement invariance

All statistical analyses were conducted with the Mplus Version 8.4 software [46]. Preliminary analyses were performed with the aim of establishing the longitudinal measurement invariance of the PVQ-21 [47]. Invariance tests were performed separately for each of the four higher order values at increasingly strict levels: configural, metric, and scalar [48]. Configural invariance was assessed by examining the overall fit of the single factor model (i.e., the self-enhancement factor was loaded by items referring to achievement and power values). Metric and scalar invariance were sequentially tested by imposing equality constraints over time on factor loadings and item intercepts, respectively. Model parameters were estimated using the maximum likelihood robust (MLR) estimation procedure. Changes in McDonald’s Non-Centrality Index (MNCI), the root mean square error of approximation (RMSEA) and the standardized root mean square residual (SRMR) fit indices between nested models were used to assess the tenability of invariance constraints. We adopted the following criteria: ΔMNCI  ≤ 0.01 [49], ΔRMSEA ≤ 0.015 [50], SRMR ≤ 0.03 and  ≤ 0.015 for metric and scalar invariance, respectively [50].

### Latent difference scores

Latent Change Scores (LCS) models, also known as Latent Difference Score models [51–54], were used to investigate value change at the intraindividual level. LCS allows modelling change across two time points as a latent factor, with its own mean and variance. Thus, interindividual differences in intraindividual change are explicitly represented in the model.

With two measurement occasions, a univariate LCS model can expressed with the following equation:

$$Y_{i},t_{2}=Y_{i},t_{1}+\Delta Y_{i};$$ 1)

The score on the construct of interest *Y* at time 2 (*Yi*,*t*_*2*_) is a function of two components: the score on *Y* at the previous time (*Yi*,*t*_*1*_) and the latent change factor (*ΔYi*), which correspond to the difference between the two measurement occasions (*Y*_*t2*_ –*Y*_*t1*_). The regression coefficients in Eq 1 (i.e., the autoregressive path and the loading from the latent change factor to *Y*_*t2*_) are fixed to 1, while the residual variance of *Yt*_*2*_ was fixed to zero. This allows to create a latent factor that captures the change between time 1 and time 2 (for a comprehensive discussion of LCS modelling, interested readers are referred to McArdle [51, 52], and McArdle and Hamagami [53]). With respect to traditional change score analyses, such as gain scores, regression residuals, and within-subjects ANOVA, this approach provides the advantage of modelling the difference between measurement occasions as true change, thus assuming that change is free from measurement error [52].

**Fig 2:**
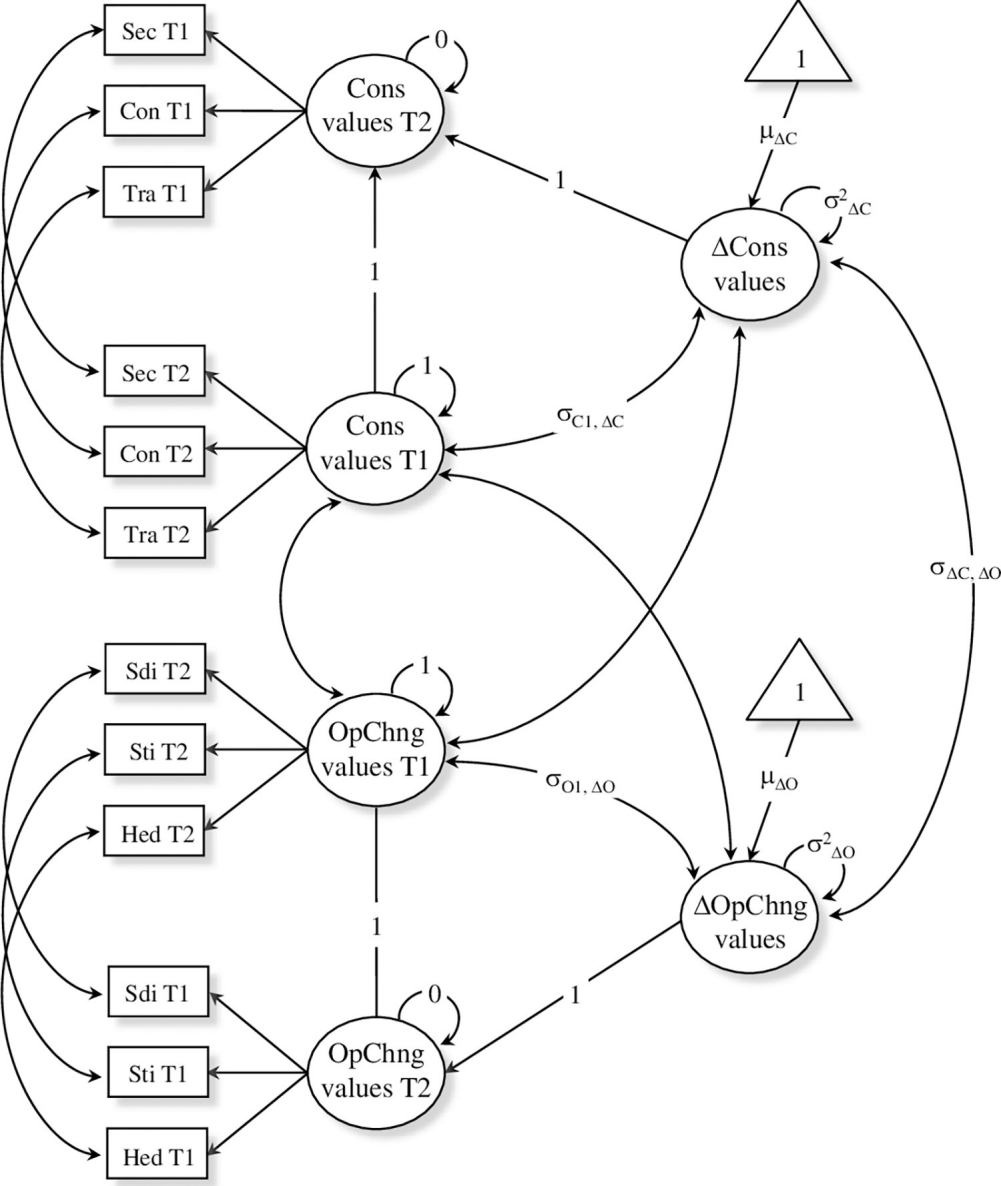
Path diagram of the unconditional LCS model for conservation and openness to change values. Sec = Security; Con = Conformity; Tra = Tradition; Sdi = Self-direction; Sti = Stimulation; Hed = Hedonism; Cons = Conservation; OpChng = Openness to Change.

In the present study, we first estimated two bivariate unconditional LCS models, each including the opposition between two competing value dimensions. The first model includes self-enhancement and self-transcendence (Model 1). The second model includes conservation and openness to change (Model 2). Values were modelled as latent constructs with multiple indicators. The ten value types were entered as uncentred (raw) scores and used as manifest variables for the respective higher order dimensions (e.g., tradition, security, and conformity were set to load on the conservation latent factor). LCS are therefore represented by second-order latent factors. As an illustrative example, the path diagram of the unconditional LCS model including conservation and openness to change values (Model 2) is represented in Fig 2.

These unconditional LCS models served to explore how values changed over the two waves of the study. Key parameters are the mean and the variance of the latent change factors. The mean reflects the average change over time. Positive means indicate an increasing importance from T1 to T2, while negative means reflect a decreasing trend. The variance captures the extent to which change in values from T1 to T2 varied across individuals (i.e., the between-subject variability). The covariance between the T1 factor and the latent change factor provides the additional information of whether the importance assigned to a given value at T1 is related to the subsequent change in value importance at T2. Finally, a parameter of interest is the covariance between the two latent change factors. This correlation reflects the dynamic interplay between conflicting values (e.g., conservation and openness to change in Model 2).

**Fig 3:**
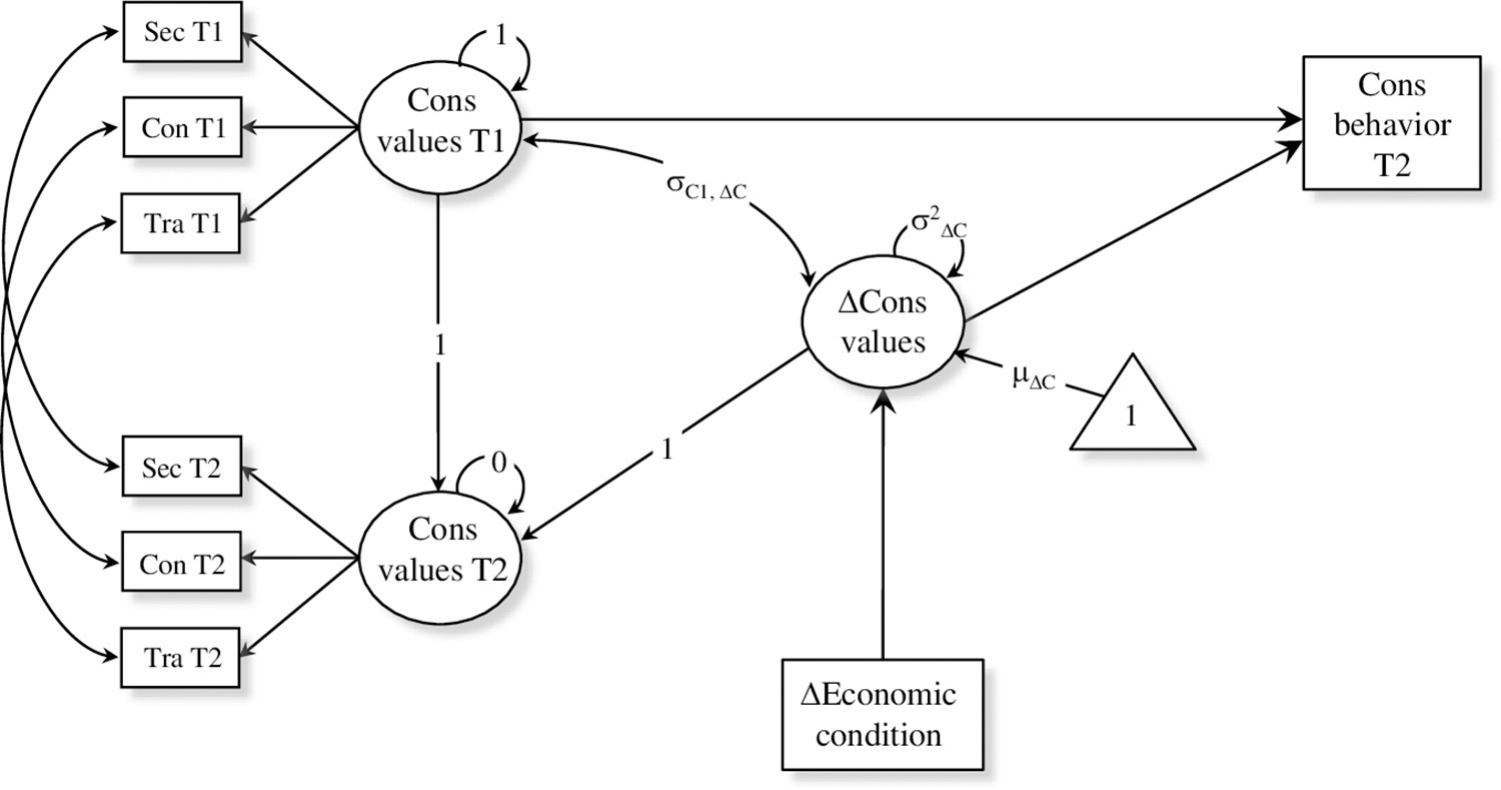
Path diagram of the conditional LCS model for conservation values. Sec = Security; Con = Conformity; Tra = Tradition; Cons = Conservation.

Next, we extended the LCS models to include both predictors and outcomes of value change. In order to account for the expected variability in the amount of change we included participants’ economic condition as a time invariant covariate affecting the latent change factors of the four higher order values. This allows to examine whether changes in values are predicted by variation in the economic status due to the pandemic. The covariate was created by comparing participants’ habitual economic condition, as it was before the pandemic, with their current condition, as reported at T2. We created a binary variable coded as 0 if the economic condition remained unchanged (*n* = 143) and 1 if it has worsened (*n* = 44). Finally, we included value-expressive behavior as outcome variables of both value importance at T1 and value change. Behaviors that express the four higher order values were included as observed variables by averaging the items of the corresponding dimensions. Missing values in the EBQ were dealt with the Full Information Maximum Likelihood (FIML) estimation method [55]. To keep the ratio between sample size and number of parameters at acceptable levels [56], the conditional LCS models were tested for each of the four higher order value separately: Self-Enhancement (Model 3), Self-Transcendence (Model 4), Conservation (Model 5), and Openness to Change (Model 6). As an illustrative example, the path diagram of the LCS model for conservation values (Model 5) is represented in Fig 3. Statistical significance was tested through the use of 95% bias-corrected confidence intervals around ML estimates, with 20000 bootstrapped replications [46].

## Results and discussions

### Descriptive statistics

Means, standard deviations, and intercorrelations among values and value-expressive behavior are reported in Table 1. Correlations were calculated using centred scores, in order to correct for individual differences in the use of the PVQ and EBQ response scales [5, 45]. The pattern of means suggests a substantial stability in values. The standardized mean change between the two waves was negligible for all the four higher order dimensions, with Cohen’s d_z_ [57] in the range of -.08 (Self-Enhancement) to .11 (Conservation). High levels of rank-order stability were also observed. Correlations over time were .78 for Self-enhancement, .71 for Self-transcendence, .77 for Conservation, and .73 for Openness to change.

**Table 1:** Descriptive statistics: Means, standard deviations, and intercorrelations among values and value-expressive behaviors.

	*M*	*SD*	1.	2.	3.	4.	5.	6.	7.	8.	9.	10.	11.	12.
1. Self-Enhancement values T1	3.32	.94												
2. Self-Transcendence values T1	5.13	.63	-.52**											
3. Conservation values T1	4.04	.65	-.35**	-.17*										
4. Openness to Change values T1	4.27	.72	-.19*	-.12	-.62**									
5. Self-Enhancement values T2	3.25	.90	.78**	-.37**	-.29**	-.17*								
6. Self-Transcendence values T2	5.14	.61	-.53**	.71**	.02	-.07	-.61**							
7. Conservation values T2	4.05	.66	-.23**	-.18*	.77**	-.46**	-.29**	-.13						
8. Openness to Change values T2	4.26	.67	-.14	.00	-.52**	.73**	-.21**	-.06	-.67**					
9. Self-Enhancement behaviors T2	2.53	.64	.40**	-.22**	-.05	-.17*	.36**	-.31**	.05	-.16				
10. Self-Transcendence behaviors T2	3.57	.74	-.29**	.37**	.03	-.05	-.27**	.45**	-.04	-.05	-.35**			
11. Conservation behaviors T2	3.06	.63	-.11	-.19*	.49**	-.26**	-.10	-.06	.50**	-.37**	-.16*	-.26		
12. Openness to Change behaviors T2	2.66	.65	.01	.02	-.37**	.38**	.02	-.08	-.42**	.48**	-.40**	-.36**	-.43**	

*Note*.

* *p* < .05

** *p* < .01.

In line with the theory [3, 17], values that are opposite in the circle showed negative and significant within time correlations: Self-enhancement and Self-transcendence values correlated -.52 (p < .01) at T1, and -.61 (p < .01) at T2. Conservation and Openness to change values correlated -.62 (p < .01) at T1, and -.67 (p < .01) at T2. Moreover, Self-enhancement and Conservation correlated negatively (r’s were -.35, p < .01, at T1, and -.29, p < .01, at T2). Correlations between Self-transcendence and Openness to change were also negative, though not significant (r’s were -.12 at T1 and -.06 at T2). These results are likewise consistent with the theory [3, 17]. Finally, Conservation correlated negatively with Self-transcendence (r’s were -.17, p < .05, at T1, and -.13, *ns*, at T2), while Openness to change correlated negatively with Self-enhancement (r’s were -.19, p < .05, at T1, and -.21, p < .01, at T2). This is a less common pattern. Both pairs of values, indeed, showed predominantly positive correlations in previous studies using the PVQ [58], although with significant variability across samples. In the meta-analytic study by Rudnev, Magun, and Schwartz [58], for example, the correlations between Conservation and Self-transcendence varied from -.45 to .55, while those between Openness to change and Self-enhancement varied from -.36 to .60. The correlations observed in the present study fall within this range. The deviation from what is commonly observed in the value literature could be ascribed, at least in part, to the context of the research. For example, the social restriction measures introduced by the Italian government might have limited the opportunities for Conservation and Self-transcendence to be pursed simultaneously. Arguably, complying with these restrictions supported the behavioral expression of conformity and security values but, to some extent, conflicted with values that express care for the welfare of others. This could explain the negative, though weak, correlation between the two value dimensions observed in the present study.

Values correlated positively with behaviors that are supposed to express compatible motivational goals, both concurrently and over time. For example, Conservation behaviors at T2 correlated .49 (p < .01) and .50 (p < .01) with Conservation values at T1 and T2, respectively. By contrast, values correlated negatively with behaviors that express motivationally opposed goals. For example, Conservation behaviors at T2 correlated -.26 (p < .01) and -.37 (p < .01) with Openness to change values at T1 and T2, respectively.

### Measurement invariance

Results of measurement invariance tests are reported in Table 2. The single factor model fit the data well for each of the higher order dimension. Thus, scalar invariance held across the two waves of the study. Metric invariance was fully supported for self-enhancement, self-transcendence, and conservation values. Partial metric invariance was established for openness to change values (ΔMNCI   = 0.014). One item (“Having a good time is important to her. She likes to “spoil” herself”) was found to be noninvariant and was allowed to vary over time. Scalar invariance was fully supported for all values. In sum, results suggest that the same constructs were measured equally over time. This a prerequisite for a meaningful interpretation of latent change over time.

**Table 2:** Tests of measurement invariance across waves.

	YB-χ^2^	df	*p*	CFI	RMSEA	SRMR	MNCI	Model Comparison	ΔCFI	ΔRMSEA	ΔSRMR	ΔMNCI
*Self-enhancement*												
1. Configural	23.250	15	.08	.980	.054	.034	.978					
2. Metric	25.848	18	.10	.981	.048	.042	.979	2 vs. 1	.001	-.006	.008	.001
3. Scalar	28.222	21	.13	.982	.043	.042	.981	3 vs. 2	.001	-.005	.000	.002
*Self-transcendence*												
1. Configural	44.119	29	.04	.949	.053	.056	.960					
2. Metric	42.964	33	.11	.967	.040	.060	.974	2 vs. 1	.018	-.013	.004	.014
4. Scalar	47.065	37	.12	.966	.038	.065	.973	3 vs. 2	-.001	-.002	.005	-.001
*Conservation*												
1. Configural	57.795	47	.13	.976	.035	.066	.971					
2. Metric	61.909	52	.16	.978	.032	.069	.974	2 vs. 1	.002	-.003	.003	.003
3. Scalar	67.942	57	.15	.976	.032	.072	.971	3 vs. 2	-.002	.000	.003	-.003
*Openness to change*												
1. Configural	95.881	47	< .001	.930	.075	.074	.877					
2. Metric	106.855	52	< .001	.921	.075	.083	.863	2 vs. 1	-.009	.000	.009	-.014
3. Partial metric	99.791	51	< .001	.930	.072	.078	.877	3 vs. 1	.000	-.003	.004	.000
4. Scalar	103.709	55	< .001	.930	.069	.077	.877	4 vs. 3	.000	-.003	-.001	.000

### Unconditional LCS models

Goodness of fit was adequate for both Model 1 (self-enhancement and self-transcendence): χ^2^(18) = 16.795, p = .54, CFI = 1.000, RMSEA = .000 (.000, .061), SRMR = .045), and Model 2 (conservation and openness to change): χ^2^(52) = 71.824, p = .04, CFI = .976, RMSEA = .045 (.012, .069), SRMR = .064. Standardized factor loadings were in the range .61 –.84 in Model 1 and .40 –.92 in Model 2. Omega reliability coefficients at T1 and T2 were respectively .76 and .69 for self-enhancement, .62 and .70 for self-transcendence, .57 and .54 for conservation, .67 and .67 for openness to change. The mean (*μ*_*Δ*_) and variance (*F073*^*2*^_*Δ*_) of the latent change scores are reported in Table 3.

**Table 3:** Structural parameters of bivariate unconditional LCS models.

	μ_*Δ*_	*σ* ^*2*^_*Δ*_	*r*_*t1*,*Δ*_
*Model 1*:			
Self-Enhancement	-.07 (-.19, .06)	.21* (.03, .49)	-.39* (-.72, -.03)
Self-Transcendence	.01 (-.14, .16)	.32* (.06, .59)	-.21 (-.54, .47)
*Model 2*:			
Conservation	.08 (-.10, .26)	.45* (.09, .91)	-.38 (-.65, .06)
Openness to change	.00 (-.12, .12)	.27* (.10, .52)	-.40* (-.62, -.09)

*Note*.

* p < .05; μ_*Δ*_ *and σ*^*2*^_*Δ*_ are the mean and the variance of the latent change scores, respectively; *r*_*t1*,*Δ*_ is the correlation between value score at T1 and value change.

The means were not significantly different from zero for all values. This suggests no reliable mean change over the two waves of the study. Of interest, the variability around the average change was statistically significant for all of the four value dimensions. Thus, individuals differed significantly in the degree to which their values changed over time. Self-Enhancement values at T1 correlated negatively (*r* = -.39, p < .05) with subsequent changes in the same values. Similar results were found for openness to change values (*r* = -.40, p < .05). This indicates that individuals with lower scores on these values at T1 increased more at T2 than those with higher scores. Negative correlations between value importance at T1 and value change were observed also for self-transcendence and conservation, but these were not statistically significant. Finally, non-significant correlations were found between latent change factors of conflicting values.

### Conditional LCS models: Exploring antecedents and consequences of value change

Table 4 reports the goodness of fit indices for the extended LCS models including predictors (economic condition) and outcomes (value-expressive behavior) of value change. Models yielded close to acceptable fit for each of the four higher order dimensions. Structural parameter estimates (i.e., standardized regression coefficients) from these models were reported in Table 5.

**Table 4:** Goodness of fit indices of univariate conditional LCS models.

	χ^2^	df	*p*	CFI	RMSEA	SRMR
*Model 3*: Self-Enhancement	5.426	7	.608	1.000	.000 (.000, .077)	.034
*Model 4*: Self-Transcendence	7.723	7	.036	.997	.023 (.000, .095)	.040
*Model 5*: Conservation	32.496	19	.028	.964	.0.62 (.021, 097)	.080
*Model 6*: Openness to Change	20.233	19	.381	.998	.019 (.000, .068)	.079

**Table 5:** Parameter estimates (standardized regression coefficients) for univariate conditional LCS models.

	Δ Economic condition → Δ Value	T1 Value → T2 Behavior	ΔValue → T2 Behavior
Model 3: Self-Enhancement	.11 (-.26, .36)	.36* (.00, .59)	-.10 (-.79, .22)
Model 4: Self-Transcendence	.09 (-.23, .39)	.54** (.26, .75)	.04 (-.45, .30)
Model 5: Conservation	.18* (.04, .35)	.80** (.47, 1.48)	.36* (.07, .97)
Model 6: Openness to Change	.06 (-.17, .35)	.81** (.62, 1.18)	.35* (.00, .80)

Note.

* p < .05

** p < .01; 95% bias-corrected bootstrap confidence intervals are reported in parenthesis.

Results showed that a decreasing economic condition led individuals to increase the importance assigned to conservation values, as reflected in the positive and significant effect of the covariate on the latent change factor (*β* = .18, p < .05). This indicates that the economic condition was able, at least in part, to explain why the importance attributed to conservation values changed more for some individuals than for others. Changing economic condition, by contrast, did not predict a shift in the importance attributed to self-enhancement, self-transcendence, and openness to change values.

As expected, we found significant effects from values to later behavior. Specifically, behaviors at T2 were positively predicted by the initial importance assigned to the values that express them: *β*’s were. 36, p < .05, for self-enhancement, .54, p < .01, for self-transcendence, .80, p < .01, for conservation, and. 81, p < .01, for openness to change. Most importantly, behaviors at T2 were additionally predicted by an increase in conservation (*β* = .36, p < .05) and openness to change (*β* = .35, p < .05) values. The same effect was not found for self-enhancement and self-transcendence values.

## Conclusions

The present study adopted a two-occasion longitudinal design to examine change in basic personal values over 100 days during the COVID-19 pandemic in Italy. Latent difference score modelling revealed no average change for the four higher order values over two different phases of the pandemic cycle. Contrary to our expectations, the importance assigned to conservation values did not increase as the pandemic became more severe. Apparently, these results deviate from those found in earlier research. The Australian study by Daniel et al. [18] reported an increase in conservation values after the beginning of the pandemic. The Polish study by Bojanowska et al. [20] observed an increase in security and conformity, but not in tradition values. Differently from these studies, however, we examined value change over a short time span. Our results might suggest that personal values are less subjected to change abruptly or in relatively short periods of time, even in the face of consistent variations in the social environment. However, in the present study values were not assessed before the COVID-19 outbreak. Therefore, we can’t exclude that study participants changed their values right after the onset of the pandemic.

Moreover, as reasoned above, mean-level stability does not imply that values have remained unchanged. An essential characteristic that informs on the process of change refers to between-person differences in how a given variable modifies over time [59]. We modelled interindividual differences in intraindividual change and found significant heterogeneity in patterns of change for all the higher order values. This suggests that individuals varied widely from each other in how their values changed over the two phases of the pandemic. Identifying systematic sources of this variability represent an important research endeavour in the study of value stability and change.

Obviously, there may be enormous differences in how people experienced the COVID-19 emergency. The effect that the pandemic event exerted on the individual’s value orientation can be potentially moderated by a number of individual and contextual factors. Daniel et al. [18], for example, have found that value change was amplified among individuals who were more worried about the pandemic. In the present study, we investigated the role of economic condition. We found that individuals who have reported a reduction in their standard of living as a result of the pandemic were more likely to increase their conservation values over the two waves of the study. This suggests that–all other conditions being equal–the pandemic exerted a greatest impact on personal values for individuals who have suffered the most serious economic consequences.

We also attempted to relate value change to value-expressive behavioral tendencies. We found that each of the four higher order value significantly predicted subsequent individual’s engagement in behaviors that express them. This result replicates what has been found in earlier studies [37, 38, 45]. Of interest, change in conservation and openness to change values also predicted behavior. In accordance with our expectations, the more these values became important, the more the individuals engaged in actions that are consistent with their fulfilment. Hence, value importance at T1 and value change have unique, additive effects on behavior. This effect, however, was not observed for self-enhancement and self-transcendence values. Change in these values was unrelated with value-expressive actions. We can only speculate about possible explanations. Verplanken and Holland [40] have suggested that value activation affect behavior only for values that are central to the self-concept. We did not have measured value centrality. Hence, this assumption cannot be tested directly. However, it is reasonable that the opposition between conservation and openness to change values may have been perceived as more salient in the second wave of the study, when the virus was widespread. A strict adherence to COVID-19 preventive measures during this pandemic phase provided protection against threats to personal safety but limited the expression of values emphasizing excitement and autonomy of action. As the literature appears to suggest, values that increased their contextual salience become more accessible [60]. This may have reinforced their effect on subsequent value-congruent behavior.

The present study is subject to several limitations. As anticipated, we did not have the possibility to collect data before the pandemic. Moreover, with only two waves of data collection we were not able to identify a trajectory of change. Including multiple waves of data would have allowed a more effective description of how values changed during the course of the pandemic. In addition, value-expressive behavior were assessed at time 2 only. This limits the possibility of making causal inferences. Although we controlled for initial levels of value importance when examining whether change in values can predict value-expressive behavior, the possibility of a reverse causation cannot be ruled out. That is, value change might have been a consequence rather than an antecedent of behavior. It should be noted, however, that whereas earlier studies have shown that values and behavior may have reciprocal influences over time, the predominant direction of causality was from the former to the latter [29, 61]. Whether this pattern replicates in the pandemic time or in the midst of other life critical events, when values are more subjected to change, is a matter of future investigation.

Another potential limitation is that participants’ economic condition, as it was before the pandemic, was measured retrospectively. Moreover, we focus on perceived, not actual economic condition. Future studies are needed to investigate whether the current results generalize to more objective measures, such as household income. Nevertheless, we believe that the way people feel about their economic condition may have important implications for value change. Earlier studies based on ESS data have indeed shown that feelings about household’s income was able to predict relevant criteria, such as religious service attendance [62], mental health [63], and life satisfaction [64]. Finally, the sample used in the present study was relatively small and not representative of the general population. This might limit the generalisability of research findings.

To conclude, the present study examined value change during two phases of the COVID-19 epidemic in Italy, characterized by different levels of intensity. While a substantial stability was found on average scores across the four higher order values, there is evidence that individuals dealing with the economic effects of the pandemic showed increased conservation values. This increase, in turn, led to act in ways that express or promote these values. If confirmed, these findings may have significant implications. Pursuing values that emphasize security, respect of social expectations, and preservation of the past may relate to various pandemic-relevant attitudes and behaviors [65, 66], as well as to a number of individual and social outcomes [67–71]. Examining how value change induced by life events is interconnected to different domains of the individual’s life, and the extent to which these connections are enduring over time, is a challenging topic for future research.

## Supporting information

S1 Table Descriptive statististic for the 24 items of the shortened version of the Everyday Behavior Questionnaire (EBQ). (DOCX)

S2 Table A two-way frequency table for feelings about economic condition before and during the pandemic. (DOCX)
