# Supplementary material for: Basic personal values in the midst of the COVID-19 pandemic in Italy: A two-wave longitudinal study
Source: PLoS One. 2022 Sep 9;17(9):e0274111. doi: 10.1371/journal.pone.0274111 (PMC9462816; doi:10.1371/journal.pone.0274111)
Supplement: S1 Table — (DOCX) [file pone.0274111.s001.docx]

**S1** **Table. Descriptive statististic for the 24 items of the shortened version of Everyday Behavior Questionnaire (EBQ).**

|  | M | SD | % never had an opportunity |
| --- | --- | --- | --- |
| 1. Pay no attention to outside pressures when making a decision (OpChng) | 3.00 | 1.25 | 1.6% |
| 2. Put a lot of energy into sports, study, music, work, etc., primarily to compete successfully (SEnh) | 3.66 | 1.25 | 1.6% |
| 3. Indulge myself by buying things that I didn’t really need (OpChng) | 2.62 | 1.28 | 2.1% |
| 4. Donate food or clothing for people in need (e.g., elderly, the homeless) (STran) | 2.49 | 1.48 | 4.3% |
| 5. Obey traffic rules even when breaking them would cause no danger (Cons) | 3.90 | 1.17 | 2.7% |
| 6. Mention to other people how valuable some of my possessions are (SEnh) | 1.22 | 0.61 | 11.2% |
| 7. Look for exciting activities to break up my routine (OpChng) | 2.13 | 1.16 | 3.2% |
| 8. Attend regular daily or weekly religious/spiritual services (Cons) | 1.77 | 1.29 | 3.2% |
| 9. Help out a friend or colleague at work or school who had a problem (STran) | 3.72 | 1.24 | 0.0% |
| 10. Try to understand the worldview of people whose beliefs about religion differed from mine (STran) | 3.32 | 1.50 | 3.7% |
| 11. Do things that provided sensual pleasure (e.g., bubble bath, massage) (OpChng) | 2.60 | 1.43 | 2.7% |
| 12. Do risky things for the thrill of it (OpChng) | 1.81 | 1.09 | 4.8% |
| 13. Plan ahead and practice hard in order to succeed at a difficult task (SEnh) | 3.54 | 1.30 | 1.1% |
| 14. Take care of a friend or family member who was sick (STran) | 3.99 | 1.28 | 0.0% |
| 15. Insist that others do what I want (SEnh) | 1.84 | 1.04 | 3.7% |
| 16. Celebrate national or ethnic group holidays (Cons) | 2.48 | 1.52 | 1.1% |
| 17. Do what I committed myself to do for a family member (STran) | 4.00 | 1.05 | 0.0% |
| 18. Take special steps so my family and I would avoid getting sick (vitamin or other supplements, wear masks, etc.) (Cons) | 4.30 | 1.02 | 0.0% |
| 19. Act independently without waiting to hear what other people were doing (OpChng) | 3.08 | 1.31 | 0.5% |
| 20. Argue for stronger government steps to preserve law and order (Cons) | 2.84 | 1.35 | 0.0% |
| 21. Study or work late into the night before an exam or project due date even though I was already well prepared (SEnh) | 2.89 | 1.46 | 2.1% |
| 22. Do what I thought was right even when someone did not approve (OpChng) | 3.38 | 1.21 | 0.5% |
| 23. Discuss suffering and poverty in the world with another person (STran) | 3.90 | 1.17 | 0.0% |
| 24. Pressure others to go along with my preferences and opinions (SEnh) | 1.87 | 1.07 | 2.7% |

*Note*. SEnh = Self-Enhancement; STran = Self-Transcendence; Cons = Conservation; OpChng = Openness to Change.
