# Supplementary material for: Basic personal values in the midst of the COVID-19 pandemic in Italy: A two-wave longitudinal study
Source: PLoS One. 2022 Sep 9;17(9):e0274111. doi: 10.1371/journal.pone.0274111 (PMC9462816; doi:10.1371/journal.pone.0274111)
Supplement: S2 Table — (DOCX) [file pone.0274111.s002.docx]

**S2 Table. A two-way frequency table for feelings about economic condition before and during the pandemic.**

|  | *Current economic condition* | | | | |
| --- | --- | --- | --- | --- | --- |
| *Before the pandemic* | 1. Living comfortably | 2. Coping | 3. Finding it difficult | 4. Finding it very difficult | Total |
| 1. Living comfortably | 56 | 16 | 0 | 1 | 73 |
| 2. Coping | 0 | 68 | 20 | 1 | 89 |
| 3. Finding it difficult | 0 | 0 | 18 | 6 | 24 |
| 4. Finding it very difficult | 0 | 0 | 0 | 1 | 1 |
| Total | 56 | 84 | 38 | 9 | 187 |
